# ARID2: A new tumor suppressor gene in hepatocellular carcinoma

**DOI:** 10.18632/oncotarget.355

**Published:** 2011-11-16

**Authors:** Hong Zhao, Jian Wang, Yongqing Han, Zhen Huang, Jianming Ying, Xinyu Bi, Jianjun Zhao, Yi Fang, Haitao Zhou, Jianguo Zhou, Zhiyu Li, Yefan Zhang, Xue Yang, Tao Yan, Linfang Wang, Michael S. Torbenson, Jianqiang Cai

**Affiliations:** ^1^ Department of abdominal surgical oncology, Cancer Hospital, Chinese Academy of Medical Sciences and Peking Union Medical College, Beijing, 100021, P.R. China; ^2^ National Laboratory of Medical Molecular Biology, Institute of Basic Medical Sciences, Chinese Academy of Medical Sciences and Peking Union Medical College, Beijing, 100005, P.R. China; ^3^ Department of Pathology, Cancer Hospital, Chinese Academy of Medical Sciences and Peking Union Medical College, Beijing, 100021, P.R. China; ^4^ Department of Pathology, Johns Hopkins University School of Medicine, Baltimore, MD 21231, USA

**Keywords:** Hepatocellular Carcinoma, Hepatitis C Virus, ARID2, Tumor Suppressor, Interferon

## Abstract

Hepatocellular carcinoma (HCC) is one of the most common malignancies worldwide, however, genetic-environmental interactions and mechanisms associated with the development of HCC remains largely unclear. Our recent work described novel inactivating mutations of *ARID2* (AT-rich interactive domain 2) in four major subtypes of HCC through exomic sequencing of ten HCV-associated HCCs and subsequent evaluation of the tumors from additional affected individuals. Here, we summarize the current knowledge about the relevance of ARID2 in HCC and the implication in future patient care.

## INTRODUCTION

Hepatocellular carcinoma (HCC) is one of the most frequent malignant diseases worldwide. With an estimated 748,000 newly diagnosed cases per year and a low five-year survival rate, HCC is the third leading cause of cancer deaths [1, 2]. Epidemiologic studies have conclusively linked viruses and chemicals to the development of HCC [3]. Among the viruses, hepatitis B (HBV) and C (HCV) viruses attribute to HCC development in more than 80% of the HCC cases [4]. The non-viral risk factors, including dietary aflatoxin B1 (AFB1) exposure, cigarette smoking and heavy alcohol consumption, can have synergistic effects [5]. Besides these risk factors, several disorders such as cirrhosis alone and hemochromatosis are associated with an increased risk of HCC [6].

In recent years, the mechanisms and genetic-environmental interactions associated with the development of HCC have been elucidated [7, 8]. Genomic and gene expression analyses have identified key dysregulated signal transduction pathways involved in liver carcinogenesis [9]. The genomic structural changes including recurrent allelic deletions and regional losses and gains have been found on several chromosomes [10-14]. Epigenetic changes in genomic DNA appear to act by directly suppressing gene expression as well as indirectly by creating conditions that increase the chance of generating hepatocyte populations containing critical combinations of structurally and functionally aberrant genes [10-12]. Several critical genes including oncogenes such as *c-myc* and *N-ras* [15] and tumor suppressor genes such as *TP53, Rb1, CDKN2A, Axin1* are located in the chromosome regions of genomic and epigenetic changes [9-11, 15, 16].

## MUTATIONS IDENTIFIED IN HCC

To gain additional insights into the genetic basis of HCC, we performed exomic sequencing for ~18,000 protein-coding genes in the cancers and normal tissues of ten individuals with HCV-associated HCC [17]. Four hundred and twenty-nine non-synonymous somatic mutations in 411 genes were identified. Five genes, which were somatically mutated in more than one tumor, were further analyzed. Among these, *CTNNB1* was mutated in four tumors, *TP53* was mutated in three tumors, and *ARID2, DMXL1* and *NLRP1* were each mutated in two tumors. The former two genes, *CTNNB1* and *TP53*, have been previously observed in HCC as tumor suppressor genes, but the other three have not been reported in any tumor type to our knowledge. The *ARID2* mutations, which seemed enriched in HCV-associated HCC in the US and European patient populations (14%, 6 out of 43 tumors) compared with the overall mutation frequency (6.5%, 9 out of 139 tumors), attracted much interest.

### Human ARID2 and its structural characteristics

ARID2 (AT-rich interactive domain 2) was initially identified in the Polybromo-associated BRG1-associated factor (PBAF) complex, a SWI/SNF chromatin-remodeling complex involved in ligand-dependent transcriptional activation by nuclear receptors [18-20]. Human ARID superfamily includes fifteen members which are classified into seven subfamilies named ARID1 through ARID5, JARID1 and JARID2 [21, 22]. ARID1 consists of two members, ARID1a and ARID1b. ARID1a, ARID1b, and ARID2, also known as BAF250a, BAF250b and BAF200, respectively, are all subunits of the SWI/SNF complexes.

**Figure 1:**
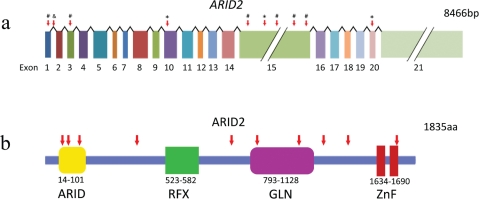
Somatic ARID2alterations identified in HCC a, Somatic alterations identified in the ARID2 gene. *, nonsense mutations; #, frame-shifting indels; &, splice site mutation. b, ARID2 protein and the inactivating alterations (red arrows) Truncating mutations are indicated by red arrows. ARID, AT-rich interaction domain; RFX, RFX-like DNA binding motif; GLN, Proline-and glutamine-rich region; ZnF, C2H2 Zinc Fingers.

The human *ARID2* gene is located on chromosome 12q and consists of 21 exons (Figure 1a). Its orthologs have been found in mouse, rat, cattle, chicken, and mosquito. The ARID2 protein contains a conservative N-terminal AT-rich DNA interaction domain (ARID), a RFX-type winged-helix, a proline- and glutamine-rich region, and two conservative C-terminal C2H2 Zn-fingers motifs (Figure 1b) [23]. ARID-containing proteins are involved in a variety of biological processes including embryonic development, cell lineage gene regulation, and cell cycle control [24]. Besides the N-terminal domain, the RFX domain is another DNA-binding domain and was named after Regulatory Factor X, a protein that binds to the X-box of the MHC class II genes [25]. The two C2H2 Zn-fingers form the tandem CWCH2 (tCWCH2) motif that is the most popular DNA-binding motif among putative eukaryotic transcription factors [26]. Recent studies regarding the binding capability of Zinc-finger domains revealed that zinc fingers can bind not only to DNA but also to RNA and protein. Therefore, it is plausible that the double Zinc-finger of ARID2 has the potential to interact with DNA, RNA, and/or proteins [27, 28].

## *ARID2*: A NEW TUMOR SUPPRESSOR GENE IN HCC

The three types of *ARID2* mutations identified in the HCV-associated HCCs are frame-shifting deletion, nonsense mutation and splice site alteration [17] (Figure 1a). These alterations are predicted to truncate and inactivate the ARID2 protein (Figure 1b). Interestingly, some of the mutations disrupt the Zn-finger motifs only, suggesting the importance of these motifs in the biological activity of ARID2.

In addition to genetic evidence, functional studies have shown that ARID2 was the only subunit in PBAF with short transcript half-life and suppression of ARID2 by small interfering RNA reduced the protein levels of other subunits in the PBAF complex [20]. Thus, ARID2 is essential for the stability of the PBAF complex. Interestingly, a recent study employing exomic sequencing has identified high-frequency (92/227, 41%) truncating mutations in the *PBRM1* gene in renal clear cell carcinomas [29]. *PBRM1* encodes the BAF180 protein, another chromatin targeting subunit of the PBAF complex. A third PBAF protein likely involved in tumor genesis is BRD7. The *BRD7* gene is frequently deleted in the breast cancers with wild-type p53 [30]. Mutations in subunits of BAF, another SWI/SNF complex, have also been found in human cancers. Inactivating mutations in *ARID1A* (BAF250a) were identified in approximately 50% of ovarian clear-cell carcinomas [31], 30% of endometrioid carcinomas [32], 83% of gastric cancers with microsatellite instability [33], 10% of colorectal cancers [34], and 19% of transitional cell carcinoma of the bladder[35]. In addition, biallelic inactivating alterations in the *hSNF5/INI1* gene which encodes a subunit shared by the PBAF and BAF complexes were found in almost all malignant rhabdoid tumors [36-38]. These observations strongly suggest that the SWI/SNF complexes have tumor-suppressing activities and *ARID2* is a tumor suppressor gene.

## ARID2 AND IFN SIGNAL TRANSDUCTION

**Figure 2:**
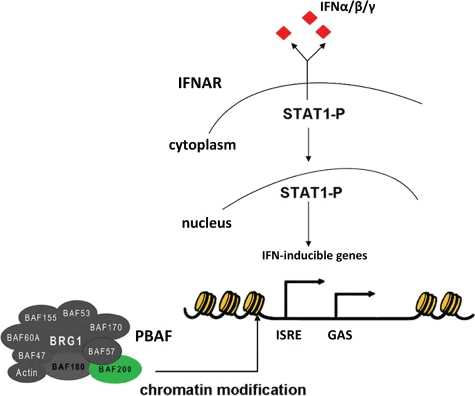
Schematic representation of the PBAF complex containing ARID2 (BAF200) which is involved in the transcriptional initiation of the human IFN-α/β/γ genes IFN, Interferon; IFNAR, Interferon-α/β/γ receptor; PBAF, Polybromo-associated BRG1-associated factor; ISRE, Interferon-sensitive response element; GAS, Interferon-γ-activated site; STAT1, Signal transducers and activators of transcription 1.

Increasing evidence suggests that the SWI/SNF complexes mediate cellular antiviral activities by binding to the IFN-inducible promoters to facilitate chromatin remodeling in response to IFN signaling [39-41] (Figure 2). A recent study has identified ARID2 as a specificity subunit in PBAF [20]. Functional analysis showed that suppression of ARID2 by small interfering RNAs specifically abolished transcription of the interferon-α-induced *IFITM1* (interferon-induced transmembrane protein 1) gene, but not the others examined. Interestingly, IFITM1 is required for the IFN-induced anti-proliferative activity in hepatocellular carcinoma cells and non-malignant hepatocytes [42]. Thus, ARID2 seems to play an important role in regulating the expression of a subset of the interferon responsive genes and in mediating the anti-proliferative activity. It is conceivable that the *ARID2* mutations abrogate the induced expression of these genes upon IFN signal transduction, which sets a stage for HCV virus and the infected host cells to escape from the IFN anti-proliferative activities. In addition, the infected host cells harboring *ARID2* mutations may have lost the ability to express higher levels of the class I MHC molecules in response to IFN signaling, making them less visible to the cytotoxic T lymphocytes [43]. These cells will then proliferate in an uncontrolled fashion and have the opportunity to acquire more genetic alterations and clonally expand into full-blown cancers.

## FUTURE STUDIES

Strong genetic and functional data have been provided to support the notion that *ARID2* is an important tumor suppressor gene in HCC. The future work should capitalize on this discovery by focusing on the following research efforts that could lead to significant improvement in patient care.

1. Clinical studies should be conducted to investigate whether *ARID2* mutations impact the prognosis of HCC patients in general and the prognosis of patients who have undergone treatment (e.g. curative therapy, IFN therapy, etc.). Adjuvant IFN therapy following curative HCC treatment has shown encouraging results, but more clinical studies are needed before it can be accepted as the standard of care for HCC patients [44-47]. Based on the observation that ARID2 was involved in IFN signaling, it is tempting to hypothesize that the *ARID2* mutations might impact the outcome of the adjuvant IFN therapy. Thus, in future studies for adjuvant IFN therapy, the resected tumors should be genotyped for *ARID2* mutations. The information obtained from these studies could be used to redirect specific health care resources to the patient populations that benefit the most.
2. We have observed an enrichment of the *ARID2* mutations in HCV-associated HCC in the US and European patient populations (6/43 tumors, 14%), compared with HBV-associated HCC in the Chinese population (1/50 tumors, 2%, P = 0.046) [16]. However, a larger study including more Chinese and Western patients with HCV-associated HCC is required to confirm the result and rule out the possibility that ethnic or environmental factors contribute to this difference. It should be noted that HCV has six major genotypes which are clustered based on geographic regions [48, 49]. For example, type 1a and 1b of HCV are dominant among the US patients, whereas type 1b and 2a are more common in China. Thus, HCV genotyping should also be performed for Chinese and Western patients to investigate whether the *ARID2* mutations are correlated with specific HCV subtypes.
3. The PBAF chromatin-remodeling complex seems to be a preferred target of tumorigenesis in both HCC and renal clear cell carcinoma. Furthermore, its specificity subunit ARID2 is required for the expression of a subset of the IFN-inducible proteins, including IFITM1 that mediates the IFN anti-proliferative activity. Studies to further characterize the biochemical and biological activities of the PBAF complex and its subunits, as well as the signal transduction pathways it regulates will provide insights that could help design novel therapeutic approaches.

HCC remains a leading cause of cancer deaths, despite worldwide efforts to develop more effective therapeutic approaches. Personalized therapy brings new hope for HCC patient care. Stratification of patients based on the genetic defects identified in the tumors could help redirect precious patient care resources to those who would benefit most and thus, reduce the health care cost. More importantly, treatment plans tailored to individual patients could result in better therapeutic response. The identification of *ARID2* as an important tumor suppressor gene in HCC provides another target that can be exploited for personalized medicine.
